# Transdiagnostic considerations of the relationship between reward sensitivity and psychopathological symptoms - a cross-lagged panel analysis

**DOI:** 10.1186/s12888-023-05139-3

**Published:** 2023-09-04

**Authors:** L. Potsch, W. Rief

**Affiliations:** Clinical Psychology and Psychotherapy, Department of Psychology, Philipps-University of Marburg, Gutenbergstr. 18, D-35032 Marburg, Germany

**Keywords:** Reward processing, Reward sensitivity, Depression, Alcohol consumption, Social anxiety, Eating disorder, Positive valence system, RDoC, Transdiagnostic

## Abstract

**Background:**

Reward sensitivity constitutes a potential key mechanism regarding the etiology and maintenance of mental disorders, especially depression. However, due to a lack of longitudinal studies, the temporal dynamics are not clear yet. Although some evidence indicates that reward processing could be a transdiagnostic mechanism of disorders, these observations could be also a product of comorbidity with depression. This study aimed at investigating the temporal dynamics of reward sensitivity and the course of psychopathological symptoms in a longitudinal investigation, while taking a possible mediating role of depression into account.

**Methods:**

We conducted a three-wave longitudinal online survey with a 4-week interval. A total of *N* = 453 participants filled out all three questionnaires. Reward sensitivity was assessed with the Positive Valence System Scale-21 (PVSS-21), depression with the Patient Health Questionnaire (PHQ-9), eating disorder symptoms with the Eating Disorder Examination-Questionnaire-8 (EDE-Q-8), social anxiety with the Mini-social phobia inventory (Mini-SPIN) and alcohol consumption with the Alcohol Use Disorders Identification Test-Consumption (AUDIT-C). Cross-lagged panels and mediation analyses were calculated using path analyses.

**Results:**

Depressive and eating disorder symptoms predicted reward insensitivity at later points in time. Effects were larger from T2 to T3. A bidirectional relationship concerning social anxiety was found. Higher alcohol consumption predicted higher reward sensitivity. Depression at T2 fully mediated the association between psychopathological symptoms at T1 and reward sensitivity at T3 for social anxiety and eating disorder symptoms.

**Conclusions:**

Our findings imply that reduced reward sensitivity seems to be a consequence rather than an antecedent of psychopathological symptoms. Comorbid depression plays a crucial role in other mental disorders regarding observed hyposensitivity towards rewards. Therefore, our results do not support a transdiagnostic notion of reward sensitivity, but they indicate a potential role of reward sensitivity for symptom persistence.

**Trial registration:**

The study was preregistered at the Open Science Framework (OSF) (https://archive.org/details/osf-registrations-6n3s8-v1; registration DOI 10.17605/OSF.IO/6N3S8).

**Supplementary Information:**

The online version contains supplementary material available at 10.1186/s12888-023-05139-3.

## Background

Reward processing plays a vital role in our daily lives. The reward system acts as a feedback loop through which we learn and adapt our behavior. A high pursuit for rewards facilitates reaching goals. However, people differ in the way they anticipate and respond to rewards [1]. A key requirement for benefiting from rewarding experiences is an adequate reward sensitivity [2]. Altered reward sensitivity and its implication for the course of psychopathological symptoms has been the focus of recent discussions [3, 4]. Reward sensitivity represents a potential crucial mechanism regarding the etiology and maintenance of psychopathological conditions [5].

The National Institute of Mental Health’s (NIMH) Research-Domain-Criteria (RDoC) framework captures reward processes within the Positive Valence System (PVS). Low reward sensitivity conceptualizes a deficit in the PVS. Moreover, the RDoC initiative encourages diverse units of analysis, e.g., behavioral, physiological, and self-report data, and supports transdiagnostic investigations of psychiatric disorders [6].

The mental illness that has been extensively researched considering reward insensitivity is depression. Depression is characterized by deficits in numerous facets of reward processing [2, 7] and entails a hyposensitivity towards rewards [5, 8, 9]. Approximately one third of people with depression suffer from anhedonic symptoms, a disruption in the appetitive reward system [10]. However, there is not only evidence for dysfunctional reward processing in depression. Some evidence indicates an aberrant transdiagnostic pattern of reward processing [5, 11]. With regards to eating disorders, studies revealed altered reward-related responses [12–16] and aberrant reward learning [17]. In self-reports, a meta-analysis found that bulimia and anorexia nervosa (binge/purge type) were associated with a hypersensitivity to rewards, although contradicting results exist for anorexia nervosa (restrictive type) [12]. Effects have even been shown independent of food-related rewards [14, 18]. Furthermore, appetitive responding seems to be reduced in anxiety, as well as depressive disorder [19]. Impairments regarding the experience of positive affect have especially been observed in social anxiety disorder [20, 21]. Patients with social phobia display a neural hyposensitivity during reward anticipation compared to healthy controls [22, 23]. Additionally, there is evidence for aberrant neural reward processing in numerous substance use disorders compared to healthy controls [24]. In another meta-analysis, an enhanced brain activation in the reward system pointed towards hypersensitivity, especially regarding drug-related stimuli [25]. A similar pattern has been observed in non-substance-related rewards [25].

When we consider studies examining the reward-related processes, the heterogeneity with regards to the assessment method is evident. Hyposensitivity towards rewards has been shown in neural and behavioral responses, as well as in self-report [4, 7, 9, 26, 27]. Meta-analyses on studies employing functional magnetic resonance imaging (fMRI) showed that disruptions of the frontostriatal circuit [9, 27] and dysregulated corticostriatal connectivity [28] are associated with deficient reward processing in depression. Similarly, deficits in hedonic capacity are related to an aberrant pattern of brain activation in regions such as the nucleus accumbens and the ventral striatum [29–31]. Event-related potentials, e.g., the feedback-related negativity (FRN) [9, 32] and the reward positivity (RewP) [33–35], are significantly altered in depression. In addition, behavioral tasks measure different stages of reward learning, including reward anticipation. Relevant indicators can be assessed by use of monetary incentive delay (MID) tasks [36], probabilistic reward tasks [37] or the effort expenditure for rewards task (EEfRT) [38].

Although the majority of studies examined neural substrates during behavioral tasks, self-report is less well researched. Nonetheless, self-report is a substantial source of information in clinical practice and represents a proxy of proposed constructs [8]. Clinicians need to rely on patients’ report and questionnaire data when it comes to treatment planning. Furthermore, previous research did not compare reward processing between diverse mental disorders [9]. Especially in light of the predominant role of depression concerning deficits in reward processing, it is vital to consider comorbidities [39]. It remains unclear whether reward sensitivity is directly related to other psychopathologies beyond depression, or whether depressive symptoms mediate the associations between reward sensitivity and other disorders.

A recent review proposes possible models for the relation between dysfunctional reward processing and depression [40]. The association investigated the most is the assumption that neural deficits in reward processing precede depressive symptoms [41–43]. When we consider theoretical frameworks, such as Lewinsohn’s depression model [44], a reduced sensitivity towards rewards, which can be perceived as a shortage of reinforcing stimuli, would likely lead to depressive symptoms. However, due to a small number of longitudinal studies, there is limited knowledge about whether reward insensitivity is an antecedent or consequence of psychopathological symptoms [40]. This limitation is especially evident when self-report and other psychopathological symptoms besides depressive symptoms are considered.

To address these gaps, our study aimed at examining the temporal dynamics between self-reported reward sensitivity and psychopathological symptoms via a longitudinal and transdiagnostic approach. We assumed that reward sensitivity predicts depressive symptoms, social anxiety, eating disorder symptoms and alcohol consumption at later points in time. In addition, we hypothesized that depressiveness mediates the relationship between reward sensitivity and social anxiety, eating disorder psychopathology, and alcohol consumption.

## Methods

This study was approved by the ethics committee at the Department of Psychology, Philipps-University Marburg (2021-25k). All participants were treated in accordance with the ethical guidelines of the German Psychological Society and provided informed consent.

### Participants

A total of 1035 individuals gave informed consent and filled out the baseline questionnaire (T1), 617 filled out the first follow-up assessment (T2), and 453 filled out the second follow-up assessment (T3). Following our preregistration, we only analyzed data of persons who participated at all three assessments (*N* = 453). Our inclusion criteria were that participants had to be at least 18 years old and needed to be German-speaking (at least native language level). Detailed sample characteristics are presented in Table S1 (see Suppl. Material). The sample was predominantly female (78.4%) and mean age was *M* = 30.3 years (*SD* = 11.18; range 18–80). 42.6% had a university degree. A substantial amount reported a lifetime diagnosis of depression (26%). Two third (66%) indicated they have never been diagnosed with a mental illness.

### Procedure

Recruitment lasted from July 2021 to December 2021. Participants were recruited via e-mail distribution lists, flyers or online posts on social media. We used the SoSciSurvey platform (https://www.soscisurvey.de/). Participants were informed about the aims of the study and procedure before providing informed consent. Respondents had the chance to win a computer tablet or one of four vouchers worth 50 Euros. After T1 participants completed T2 four weeks later (max. +1 week), and T3 another four weeks later (max. +1 week). Mean time for completing the survey was *M* = 15.43 min (*SD* = 6.23 min) for T1. Except for the demographics, each questionnaire was assessed three times. After the initial invitation, we reminded participants twice every two days via e-mail to participate.

### Measures

#### Demographic variables

Participants provided basic demographic information and answered questions concerning their mental health, treatment experience and the COVID-19 pandemic (see Suppl. Material, Table S1).

#### Positive Valence System Scale-21 (PVSS-21)

In accordance with guidelines [45], the PVSS-21 [4] was translated into German. Two clinical psychologists, one of whom is bilingual, translated the PVSS-21 into German. The translation was reviewed and consensually approved into one version. Next, the final German questionnaire was back translated by another team of clinical psychologists. The PVSS-21 consists of 21 items, which assess the Positive Valence System domain of the RDoC. The questionnaire measures reward responses to various reward types, which form seven subscales (Food, Physical Touch, Outdoors, Positive Feedback, Hobbies, Goals, Social Interactions; e.g., “I expected to enjoy being hugged by someone I love.”). The items map on PVS constructs (reward expectancy, reward anticipation, initial responsiveness, reward satiation, effort valuation, reward valuation) and are rated on a 9-point Likert scale from 1 (*extremely untrue of me*) to 9 (*extremely true of me*). The PVSS-21 has strong factorial validity, retest reliability, as well as good convergent validity. In addition, due to the strong connection between depression and positive valence processes, the authors of the scale evaluated the PVSS-21 in a sample of participants with and without depression. Although the PVSS-21 discriminated depressed from nondepressed individuals, it was not redundant with depressive symptoms (*r* = − .48 to *r* = − .37) [4]. Cronbach’s alpha coefficient ranged between *α* = 0.91 and *α* = 0.95. Internal consistency in the present study was *α*_T1_ = 0.912, *α*_T2_ = 0.933 and *α*_T3_ = 0.941.

#### Patient Health Questionnaire (PHQ-9)

The German version of the PHQ-9 [46] was used to measure depressive symptoms experienced in the last two weeks. The questionnaire consists of nine items that are based on the diagnostic criteria of depression from DSM-IV (e.g., “Feeling down, depressed, or hopeless”). Items are rated on a 4-point scale ranging from 0 (*not at all*) to 3 (*nearly every day*). Excellent internal reliability and validity are reported by numerous studies [46–49]. Cronbach’s alpha in the present study was *α*_T1_ = 0.875, *α*_T2_ = 0.891 and *α*_T3_ = 0.892.

#### Alcohol Use Disorders Identification Test-Consumption (AUDIT-C)

To assess alcohol consumption, we used the AUDIT-C [50]. The AUDIT-C is a brief screening tool that consists of the first three items of the 10-item AUDIT [51]. Items assess the frequency and dose of alcohol consumption (e.g., “How often do you have a drink containing alcohol?”). Questions are rated on a 4-point scale ranging from 0 to 4 (e.g., Item 1: from “*never” to “4 or more times a week”)*. The AUDIT-C is a valid measure with good to excellent psychometric properties and a high validity [52, 53]. Studies found a Cronbach’s *α* of 0.75 for the AUDIT-C and a test- retest reliability of 0.93 [54]. Cronbach’s alpha in our sample was *α*_T1_ = 0.677, *α*_T2_ = 0.699 and *α*_T3_ = 0.728.

#### Eating Disorder Examination-Questionnaire-8 (EDE-Q-8)

The German version of the EDE-Q-8 [55] is a short form of the EDE-Q [56] and assesses global eating disorder symptoms in the last 28 days (e.g., “Have you had a strong desire to lose weight?”). It consists of four subscales (restraint, eating concern, weight concern, shape concern). Items are rated on a scale of 0 (*characteristic was not present*) to 6 (*characteristic was present every day* or *in extreme form*). The EDE-Q-8 is a reliable and valid screening tool with excellent psychometric properties [55]. In the present study, the internal consistency was *α*_T1_ = 0.929, *α*_T2_ = 0.933 and *α*_T3_ = 0.942.

#### Mini - social phobia inventory (Mini-SPIN)

To assess social anxiety, we used the Mini-SPIN [57], a screening tool for social anxiety consisting of three items (e.g., “Fear of embarrassment causes me to avoid doing things or speaking to people.”). Participants report difficulties in the respective area within the last week. Statements are rated on a scale of 0 (*not at all*) to 4 (*extremely*). The Mini-SPIN was found to be a reliable brief measure with good convergent and discriminant validity [57, 58]. In our sample, the internal consistency was *α*_T1_ = 0.767 at T1, *α*_T2_ = 0.791 and *α*_T3_ = 0.793 at T3.

#### Data preparation and statistical analyses

To estimate the required sample size, we used the pwrSEM v0.1.2 application [59]. We determined that, in order to reach a minimum power of 0.90 with 1000 simulations, our sample size needs to be at least *N* = 350. Data was analyzed using IBM SPSS 27 and SPSS AMOS version 28.0.0. We checked all scales for univariate outliers. Using Mahalanobis’ distance and studentized deleted residuals, we screened data for multivariate outliers. Identified outliers were examined using Cooks’ distance to evaluate the impact. After conducting sensitivity analyses, we did not identify any influential data point and therefore did not exclude any of these cases. None of the variables deviated substantially from normality (skewness < 2, kurtosis < 7; 60). We inspected bivariate scatterplots and intercorrelations between all assumed relationships. All reported parameters are standardized coefficients. Cross-lagged panels were conducted using path analyses. Calculations were based on maximum likelihood estimation and bootstrapped confidence intervals were applied (*N* = 500, 95% confidence interval (CI)). Bivariate correlations between residuals at T2 and T3 were allowed since they are theoretically plausible. Mediations were tested using path analyses. Specific indirect effects were tested for statistical significance using bootstrapping (*N* = 500, 95% CI). For exploratory purposes, we tested the mediations in the other direction as well.

## Results

### Descriptive statistics and correlational analyses

In the present study, the mean PHQ-9 sum scores indicated mild symptoms of depression (*M*_*T1*_ = 8.66 (*SD* = 5.63); *M*_*T2*_ = 8.78 (*SD* = 5.65); *M*_*T3*_ = 8.65 (*SD* = 5.81)) [46]. PVSS-21 scores ranged between *M*_*T1*_ = 6.47 (*SD* = 1.26) and *M*_*T2*_ = 6.38 (*SD* = 1.37) (see Table 1). Most variables showed substantial cross-sectional and longitudinal relationships and were stable over the three time points (see Suppl. Material, Table S2, for detailed results). The highest bivariate correlation was found between the PVSS-21 and the PHQ-9 (e.g., *r* = − .467, *p* < .001), the lowest association showed the PVSS-21 and the AUDIT-C (e.g., *r* = .072, *p* = .126). In addition, the association between the PHQ-9 and the EDE-Q-8 (e.g., *r* = .461, *p* < .001) and the Mini-SPIN (e.g., *r* = .510, *p* < .001) were substantial. Only the AUDIT-C revealed little to no significant association with the PHQ-9 (e.g., *r* = − .003, *p* = .945).

**Table 1:** Means and Standard Deviations of the Main Variables

Variable	T1, *M* (*SD*)	T2, *M* (SD)	T3, *M* (*SD*)
PVSS-21^a^	6.47 (1.26)	6.38 (1.37)	6.41 (1.41)
PHQ-9^b^	8.66 (5.63)	8.83 (5.88)	8.65 (5.81)
EDE-Q-8^a^	2.00 (1.68)	1.98 (1.66)	1.92 (1.70)
Mini-SPIN^b^	5.16 (2.95)	5.28 (2.99)	5.22 (3.05)
AUDIT-C^b^	5.57 (2.16)	5.59 (2.21)	5.55 (2.29)

*Note: N* = 453, *M* = Mean, *SD* = Standard deviation, T1 = baseline, T2 = 4 weeks follow-up, T3 = 8 weeks follow-up, PHQ-9 = 9-item Public Health Questionnaire (module for depression), EDE-Q-8 = 8-item Eating Disorder Examination Questionnaire, Mini-SPIN = Short form of the Social Phobia Inventory, AUDIT-C = Alcohol Use Disorders Identification Test-Consumption, PVSS-21 = 21-item Positive Valence System Scale

^a^Please note that we report mean scores

^b^Please note that we report sum scores

#### Cross-lagged panels

**Fig. 1:**
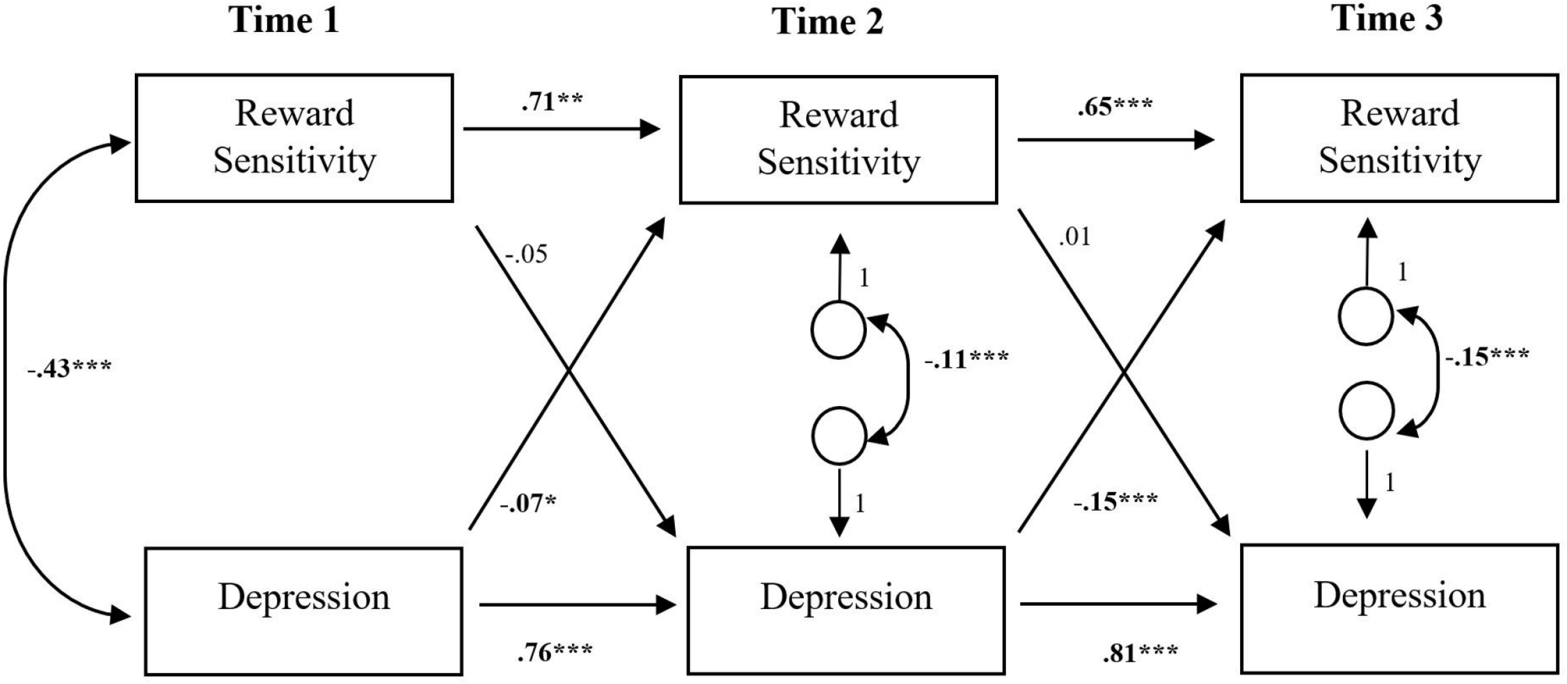
Cross-Lagged Panel With Reward Sensitivity and Depression (T1, T2, T3) *Note. N* = 453. Standardized path coefficients are reported. Reward Sensitivity was measured with the PVSS-21. Depression was measured with the PHQ-9. *p < .05. **p < .01 ***p < .001

**Fig. 2:**
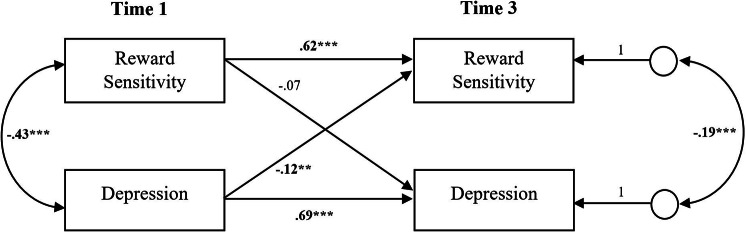
Cross-Lagged Panel With Reward Sensitivity and Depression (T1, T3) *Note. N* = 453. Standardized path coefficients are reported. Reward Sensitivity was measured with the PVSS-21. Depression was measured with the PHQ-9. *p < .05. **p < .01 ***p < .001

Reward sensitivity_T1_ did not significantly predict depression_T2_, whereas depression_T1_ showed a significant negative effect on reward sensitivity_T2_ (β = − 0.073, *SE* = 0.035, *p* = .039, 95% CI [-0.151, 0.005]). This relationship was also evident from T2 to T3. Reward sensitivity_T2_ did not significantly predict depression_T3_, however depression_T2_ showed a significant negative effect on reward sensitivity_T3_ (β = − 0.154, *SE* = 0.036, *p* < .001, 95% CI [-0.264, -0.068]). A similar pattern revealed from T1 to T3. Reward sensitivity_T1_ did not significantly predict depression_T3_, however depression_T1_ substantially predicted reward sensitivity_T3_ (β = − 0.118, *SE* = 0.038, *p* = .002, 95% CI [-0.210, -0.027]) (see Figs. 1 and 2, and Suppl. Material, Table S3, for detailed results).

Reward sensitivity_T1_ significantly predicted social anxiety_T2_ (β = − 0.065, *SE* = 0.031, *p* = .034, 95% CI [-0.123, -0.002]), whereas social anxiety_T1_ did not show a significant effect on reward sensitivity_T2_. The reverse effect was found for T2 and T3. Reward sensitivity_T2_ did not significantly predict social anxiety_T3_, however social anxiety_T2_ predicted reward sensitivity_T3_ (β = − 0.108, *SE* = 0.034, *p* = .001, 95% CI [-0.201, -0.030]). From T1 to T3 we observed a bi-directional pattern. Reward sensitivity_T1_ had a negative effect on social anxiety_T3_ (*β* = − 0.083, SE = 0.034, p = .013, 95% CI [-0.144, -0.024]), social anxiety_T1_ negatively predicted reward sensitivity_T3_ as well (*β* = − 0.097, *SE* = 0.036, *p* = .007, 95% CI [-0.175, -0.025]) (see Suppl. Material, Table S4, Figures S1, S2, for detailed results).

Neither reward sensitivity_T1_ predicted eating disorder symptoms_T2_, nor did eating disorder symptoms_T1_ predict reward sensitivity_T2_. At a later point in time, eating disorder symptoms_T2_ showed a negative effect on reward sensitivity_T3_ (β = − 0.101, *SE* = 0.033, *p* = .002, 95% CI [-0.179, -0.030]). However, reward sensitivity_T2_ did not show any significant effect on eating disorder symptoms_T3_. A similar pattern was evident from T1 to T3. Reward sensitivity_T1_ did not significantly predict eating disorder symptoms_T3_, however eating disorder symptoms_T1_ substantially predicted reward sensitivity_T3_ (β = − 0.10, *SE* = 0.036, *p* = .005, 95% CI [-0.180, -0.021]) (see Suppl. Material, Table S5, Figures S3, S4, for detailed results).

None of the cross-lagged panels between reward sensitivity and alcohol consumption achieved statistical significance, except for the relations from T1 to T3. Alcohol consumption_T1_ showed a positive effect on reward sensitivity_T3_ (β = 0.078, *SE* = 0.035, *p* = .026, 95% CI [0.012, 0.141]), but reward sensitivity_T1_ did not significantly predict alcohol consumption_T3_ (see Suppl. Material, Table S6, Figures S5, S6, for detailed results).

#### Mediation analyses

**Fig. 3:**
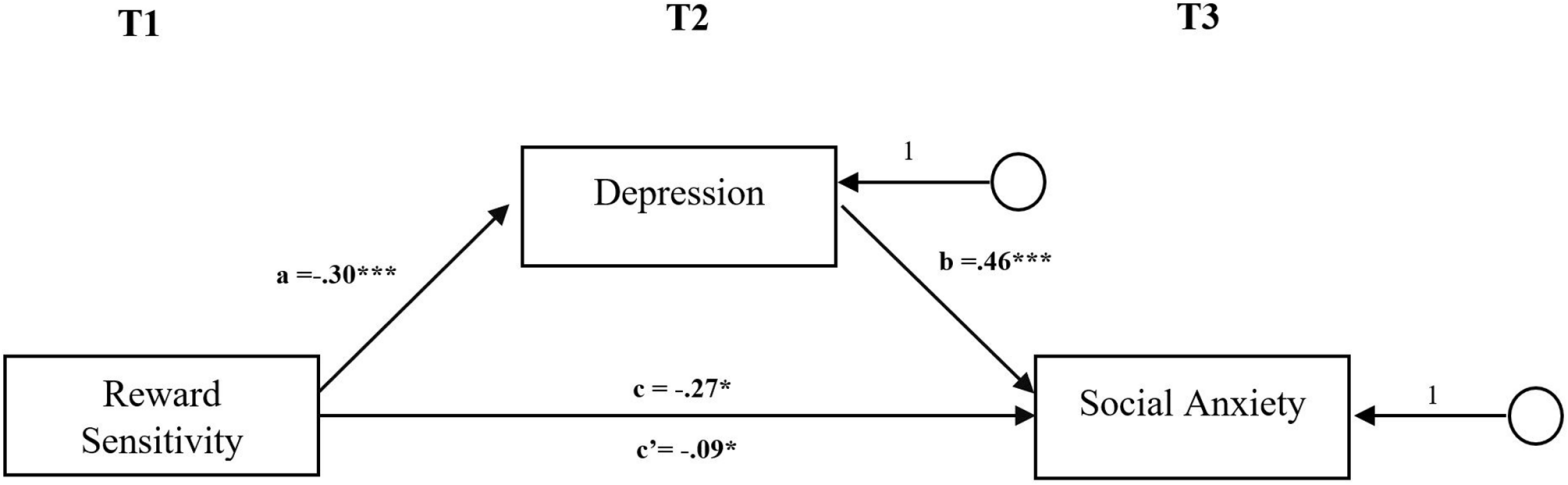
Mediation Model of Reward Sensitivity (T1) on Social Anxiety (T3) *Note. N* = 453, Standardized path coefficients are reported. Social Anxiety was measured with the Mini-SPIN. Depression was measured with the PHQ-9. Reward Sensitivity was measured with the PVSS-21. *p < .05. **p < .01 ***p < .001

As reward sensitivity_T1_ did not predict eating disorder symptoms_T3_ and alcohol consumption_T3_, most of the proposed mediations were not tested [61, 62]. Results of the proposed mediation that we were able to investigate in social anxiety are displayed in Fig. 3. An effect of reward sensitivity_T1_ on social anxiety_T3_ was observed, (*b* = - 0.267, *p* < .01). Reward sensitivity_T1_ predicted the mediator depression_T2_ significantly (*b* = -0.303, *p* < .001), which in turn predicted social anxiety_T3_ significantly (*b* = 0.464, *p* < .001). After entering the mediator into the model, the indirect effect of reward sensitivity_T1_ on social anxiety_T3_ was significant (*b* = − 0.177, *p* < .01), whilst the direct effect of reward sensitivity_T1_ on social anxiety_T3_ remained significant as well (*b* = − 0.090, *p* < .05). This indicates a partial mediation of depression_T2_ on the relationship between reward sensitivity_T1_ and social anxiety_T3_.

#### Exploratory analyses

For other variables of interest, explorative mediation models were calculated regarding the possible mediating effect of depression_T2_ on the relationship between social anxiety_T1_/eating disorder symptoms_T1_/alcohol consumption_T1_ and reward sensitivity_T3_ (see Suppl. Material, Figures S7 – S9, for detailed results).

An effect of social anxiety_T1_ on reward sensitivity_T3_ was observed (*b* = − 0.26, *p* < .01). Social anxiety_T1_ predicted the mediator depression_T2_ (*b* = 0.47, *p* < .001), which in turn predicted reward sensitivity_T3_ (*b* = − 0.401, *p* < .001). The indirect effect of social anxiety_T1_ on reward sensitivity_T3_ was significant (*b* = − 0.188, *p* < .01), whilst the direct effect of social anxiety_T1_ on reward sensitivity_T3_ was not significant anymore (*b* = − 0.077, *p* = .107). This suggests a full mediation of depression_T2_ on the relationship between social anxiety_T1_ and reward sensitivity_T3_.

We observed an effect of eating disorder symptoms_T1_ on reward sensitivity_T3_ (*b* = − 0.241, *p* < .01). Eating disorder symptoms_T1_ predicted the mediator depression_T2_ (*b* = 0.437, *p* < .001), which in turn predicted reward sensitivity_T3_ (*b* = − 0.410, *p* < .001). The indirect effect of eating disorder symptoms_T1_ on reward sensitivity_T3_ was significant (*b* = − 0.179, *p* < .01), whilst the direct effect of eating disorder symptoms_T1_ on reward sensitivity_T3_ was not significant anymore (*b* = − 0.062, *p* = .188). We found that the association between eating disorder symptoms_T1_ and reward sensitivity_T3_ was fully mediated by depression_T2_.

An effect of alcohol consumption_T1_ on reward sensitivity_T3_ was observed (*b* = 0.150, *p* < .01). However, alcohol consumption_T1_ did not predict the proposed mediator depression_T2_ (*b* = − 0.038, *p* = .414), which however in turn predicted reward sensitivity_T3_ significantly (*b* = − 0.432, *p* < .001). The indirect effect of alcohol consumption_T1_ on reward sensitivity_T3_ was not significant (*b* = 0.017, *p* = .503), whilst the direct effect of alcohol consumption_T1_ on reward sensitivity_T3_ was still significant (*b* = 0.133, *p* = .001). As a result, we found that the relationship between alcohol consumption_T1_ and reward sensitivity_T3_ was not mediated by depression_T2_.

In addition, in order to see whether the presence or absence of a history of depression was a bias, we conducted an exploratory cross-lagged panel with *N* = 118 participants who indicated that they had already suffered from depression via self-report. Depression_T1_ showed a significant negative effect on reward sensitivity_T2_ (β = − 0.147, *SE* = 0.062, *p* = .019, 95% CI [-0.297, 0.020]). A similar relationship was evident from T2 to T3 and from T1 to T3, although non-significant. For detailed results see Suppl. Material, Table S7 and Figures S10 and S11.

## Discussion

This three-wave cross-lagged panel with a four-week interval between assessment points aimed at investigating the temporal relations between reward sensitivity and psychopathological symptoms (depression, social anxiety, eating disorder symptoms, alcohol consumption). To account for possible comorbid depressive symptoms, we tested the mediating effects of depression.

Our data revealed a strong association of reward sensitivity and depression, which is in line with evidence of a recent cross-sectional review [8]. It is important to note that the cross-sectional effects of depressive symptoms on reward sensitivity appeared to be greater than the longitudinal effects. Previous literature has seldomly explored longitudinal associations, even regarding similar concepts such as anhedonia.

Contrarily to our assumed direction of the effects, depression predicted reward insensitivity at later points in time, especially from T2 to T3. In addition, an exploratory analysis revealed similar tendencies with a subsample of participants with a history of depression. Depressive symptoms predicted reward insensitivity, especially from T1 to T2. However, regarding the longitudinal traces of depression at other time points (T1 to T3 and T2 to T3), the relationship appeared non-significant and only presented a trend towards statistical significance. Nonetheless, these results should be interpreted with caution, as participants indicated a self-reported subjective lifetime diagnosis of depression. Contrary to the direction of effects in our findings, prospective studies revealed that blunted neural response to rewards predicted the onset of depression [63] and an increase in depressive symptoms in adolescents [42, 43]. Yet, it is possible that neural indices yield different results than self-report data. Accordingly, the authors of a recent review [40] confirmed low associations between behavioral tasks and self-report [64]. Of note, results between studies that use diverse assessment methods should be compared with caution. In line with our observations, the review points out that reward processing abnormalities do not provide enough evidence for the clinical prediction of depression [40].

In the case of social anxiety, we found a bidirectional relation that was respectively stronger from T2 to T3 than from T1 to T2. High associations between social anxiety and reward insensitivity are in line with cross-sectional studies revealing that social anxiety is related to an attenuated neural reactivity in anticipation of rewards [22, 23] and decreased brain connectivity during reward trials [65]. In theory, a bidirectional effect seems conclusive. Avoidance of social rewards as a symptom of social anxiety can increase hyposensitivity towards rewards. Exposure to such rewards is rare and is not characterized by active seeking of rewards, but by fear. In turn, reduced reward sensitivity, especially during socially rewarding experiences, could amplify symptoms [66]. Decreased positive experiences in social phobia [20, 67] and emerging social anhedonia [68] can be consequences.

Reward sensitivity did not predict eating disorder symptoms over time. However, eating disorder symptoms significantly predicted reward sensitivity from T2 to T3 and T1 to T3. The association between altered reward processing and eating disorder symptoms is consistent with previous research [12, 17]. Comparable to other studies [14, 18], these associations were independent of solely food-related rewards. The direction of effects is convergent with studies showing a reward hyposensitivity [12]. However, some investigations indicate a hypersensitivity towards reward [14, 15]. As outlined in preliminary work, this could owe to differences regarding subtypes of eating disorder [12].

In contrast to our initial hypothesis, reward sensitivity did not predict alcohol consumption over time. It is noteworthy that alcohol consumption was not strongly associated with reward sensitivity, a possible explanation for why there were no significant results in the first cross-lagged panel (T1, T2, T3). These results are in contrast to prior research that demonstrated aberrant reward processing in substance use disorders [11, 24, 25, 69]. However, this work mostly considered clinical samples with chronic conditions. Alcohol consumption, which was meant to serve as a proxy for alcohol-related misuse or dependence [50], could have been too unspecific and not chronic in our subclinical sample. Furthermore, previous studies mostly employed behavioral or neural indicators, and self-report questionnaires were merely considered. However, the tendency that higher alcohol consumption predicted higher reward sensitivity was shown in our second cross-lagged model (T1, T3). Although our initially proposed direction of the effects did not yield significance, our results are in line with one longitudinal study. Self-reported reward sensitivity did not predict relapse in pathological gamblers [70].

Concerning the hypothesized mediation, we found that in the case of social anxiety disorder, there was a partial mediation of depression on the relationship between reward sensitivity and social anxiety. In contrast, the association between alcohol consumption and reward sensitivity was not mediated by depression. Our exploratory analysis demonstrated that the effects of social anxiety and eating disorder symptoms on reward insensitivity were fully mediated via depression. These results were confirmed by a meta-analytic structural equation model demonstrating that reward sensitivity distinguishes anxiety from depression, as reward sensitivity only predicted depression, but not anxiety [8]. Since these results were cross-sectional, our three-wave cross-lagged design adds substantially to this finding [42, 71–73].In light of our results, we cannot preclude that the effects in the cross-lagged panels of social anxiety and eating disorder symptoms were mainly driven by comorbid depressive symptoms.

Seeing that our results were most robust with regard to depressive symptoms, this corroborates the assumption that reward insensitivity is specifically related to anhedonia [42, 71–73], a feature especially prone and specific to depression. Anhedonia can be defined as a concept based on reward insensitivity and entails strong links to reward processing in general [74]. This is why it is noteworthy to link our observations with evidence regarding anhedonia. Yet, previous literature does not fully answer the question regarding direction of effects of anhedonic and depressive symptoms either. Another study that explored the longitudinal relationship between anhedonia and depressed mood in adolescents found a bidirectional association with no apparent temporal sequence [75]. However, anhedonia represents a negative prognostic factor for pharmacological [76, 77], as well as psychological treatment [19, 78]. Another study found that patients with high anhedonia showed impaired reward learning, which in turn increased the probability of a persisting diagnosis of depression [79]. Accordingly, anhedonia could be a maintaining factor for the course of depression. This conclusion is corroborated by results of a recent meta-analysis on 12 longitudinal analyses [80]. The authors found that dampening responses to positive affect, which are a characteristic of anhedonia and specifically relate to reward insensitivity, are a risk factor for the development of depression. Nonetheless, a mutual association was also found, with baseline depression predicting tendencies to engage in dampening.

### Limitations and strengths

The current study bears several limitations. To promote a dimensional approach, we mainly focused on an unselected subclinical sample. Therefore, we cannot generalize our findings to clinical samples. For adequate diagnostics, future studies should use structured clinical interviews and implement scales that determine the severity of mental disorders more precisely. In addition, with respect to some sociodemographics (e.g., race, education, gender), our sample was relatively homogeneous. Despite the methodological strengths of the cross-lagged panel, the design is not suitable for separating stable between-person differences from within-person processes [81]. Moreover, the detected effect sizes in our models were low and scores were relatively stable over time. The assessment of reward sensitivity was solely reliant on self-report. This implies some limitations, such as that answers may have been affected by response styles [82–85]. In line with the idea of RDoC, future studies should consider assessing reward sensitivity with diverse modalities. Combining self-report with neural and behavioral indicators while exploring the congruence between different kinds of data, which are assumed to measure the same concepts, could be insightful. For instances, low reward sensitivity could be more precisely reflected by dysregulated transmitter systems or aberrant brain activation [30, 31, 86] than via self-report. The present study’s cross-lagged design with three waves of measurement constitutes an important strength [87], as there is a lack of longitudinal research in this field. In addition, the transdiagnostic consideration of reward sensitivity in a subclinical sample has been neglected in earlier studies, a gap we tried to fill in our investigation. Our longitudinal mediation analyses add substantially to cross-sectional approaches, which tend to generate biased estimates on causal processes that develop over time [88]. Another strength of the conducted research is the assessment of reward sensitivity with the PVSS-21, a robust predictor of anhedonia that partly overlaps with depressive symptoms. Since the questionnaire maps onto aspects of the RDoC, it is suitable for transdiagnostic research, which has drawn considerable attention in recent years. The PVSS-21 is ecologically valid because it measures responses to everyday life rewards and was developed to detect state changes fluctuating over time.

## Conclusion

Our findings suggest that reduced self-reported reward sensitivity seems to be rather a consequence than an antecedent of current depressive symptoms. Also symptoms of social anxiety and eating disorder predict low reward sensitivity at a later point in time. However, these relations are fully mediated via depressive symptoms and thus do not support a transdiagnostic notion of reward sensitivity. Nonetheless, more longitudinal research and replications are needed to prove the robustness of these results, especially because the cross-sectional effects have consistently been stronger than the longitudinal effects. Our analyses provide support that blunted reward sensitivity contributes to the downstream effects of depression [40], nonetheless these tendencies must not be causal. Future cross-lagged panels should explore the research question in clinical samples with special consideration of the role of anhedonia. Especially because the traces of depression have consistently been stronger from T2 to T3, future research should examine whether the effects get stronger with higher chronicity of psychopathological symptoms. Subsequent research should also consider longer time frames. Finally, interventions in psychotherapy should specifically target reward insensitivity in order to prevent chronic depression. Comorbid symptoms of depression play a crucial role regarding observed reward insensitivity in other mental disorders. Patients with diminished reward sensitivity are at risk for a persisting depression because they experience a reduced capacity to pursue and react to rewards [5, 40]. In sum, our results contribute to an enhanced understanding of a possible maintenance or chronic developments of depressive symptoms.

### Electronic supplementary material

Below is the link to the electronic supplementary material.

Supplementary Material 1: Transdiagnostic considerations of the relationship between reward sensitivity and psychopathological symptoms - a cross-lagged panel analysis

## Data Availability

On reasonable request, the analyzed dataset in the conducted study is available from the corresponding author.

## Abbreviations

NIMH: National Institute of Mental Health’s
RDoC: Research-Domain-Criteria
PVS: Positive Valence System
fMRI: functional magnetic resonance imaging
FRN: feedback-related negativity
RewP: reward positivity
MID: monetary incentive delay
EEfRT: effort expenditure for rewards task
PVSS-21: 21-item Positive Valence System Scale
OSF: Open Science Framework
PHQ-9: 9-item Patient Health Questionnaire
AUDIT-C: Alcohol Use Disorders Identification Test-Consumption
EDE-Q-8: 8-item Eating Disorder Examination-Questionnaire
Mini-SPIN: Mini - social phobia inventory
