# Supplementary material for: Transdiagnostic considerations of the relationship between reward sensitivity and psychopathological symptoms - a cross-lagged panel analysis
Source: BMC Psychiatry. 2023 Sep 4;23:650. doi: 10.1186/s12888-023-05139-3 (PMC10478275; doi:10.1186/s12888-023-05139-3)
Supplement: Supplementary file 1 — Supplementary Material 1: Transdiagnostic considerations of the relationship between reward sensitivity and psychopathological symptoms - a cross-lagged panel analysis [file 12888_2023_5139_MOESM1_ESM.docx]

# **Supplementary Material**

**Table S1** Sample Characteristics

| **Variable** | M (SD) | % |
| --- | --- | --- |
| Age | 30.3 (11.18) |  |
| Gender |  |  |
| female | -- | 78.4 |
| male | -- | 21.2 |
| diverse | -- | 0.4 |
| Education |  |  |
| Elementary | -- | 0.2 |
| High school | -- | 9.1 |
| College entrance certificate | -- | 48.1 |
| University degree | -- | 42.6 |
| Self-reported lifetime diagnoses**^a^** |  |  |
| Depression | -- | 26 |
| Social phobia | -- | 7.5 |
| Eating disorder | -- | 9.1 |
| Alcohol use disorder | -- | 0.7 |
| Other | -- | 10.2 |
| None | -- | 66 |
| Self-reported treatment |  |  |
| Previous psychiatric/psychological | -- | 41.1 |
| Current psychiatric/psychological | -- | 18.1 |
| Current psychiatric medication | -- | 9.3 |
| COVID-19 pandemic: fewer positive activities? |  |  |
| Strongly disagree | -- | 7.5 |
| Rather disagree | -- | 17.4 |
| Rather agree | -- | 53.6 |
| Strongly disagree | -- | 21.4 |

*Note. N* =453.

^a^Percentages do not add up to 100% because multiple answers were possible.

**Table S2** Intercorrelations of Main Variables at T1, T2 and T3

|  | **PVSS-21**  **(T1)** | **PVSS-21**  **(T2)** | **PVSS-21**  **(T3)** | **PHQ-9**  **(T1)** | **PHQ-9**  **(T2)** | **PHQ-9**  **(T3)** | **EDE-**  **Q-8**  **(T1)** | **EDE-**  **Q-8**  **(T2)** | **EDE-**  **Q-8**  **(T3)** | **AUDIT**  **-C**  **(T1)** | **AUDIT**  **-C**  **(T2)** | **AUDIT**  **-C**  **(T3)** | **Mini-SPIN**  **(T1)** | **Mini-SPIN**  **(T2)** | **Mini-SPIN**  **(T3)** |
| --- | --- | --- | --- | --- | --- | --- | --- | --- | --- | --- | --- | --- | --- | --- | --- |
| PVSS  -21 (T1) | - | .717*** | .663*** | -.391** | -.367*** | -.367*** | -.196*** | -.223*** | -.193*** | .094* | .072 | .106* | -.269*** | -.264*** | -.274*** |
| PVSS  -21 (T2) | - | - | .728*** | -.338*** | -.399*** | -.337*** | -.205*** | -.194*** | -.194** | .127** | -.094* | .103* | -.226*** | -.280*** | -.276*** |
| PVSS  -21 (T3) | - | - | - | -.340*** | -.401*** | -.467*** | -.231*** | -.253*** | -.253*** | .134** | .098* | .130** | -.264*** | -.292*** | -.309*** |
| PHQ-9  (T1) | - | - | - | - | .766*** | .705*** | .446*** | .434** | .395*** | -.068 | -.054 | -.102* | .493*** | .457*** | .427*** |
| PHQ-9  (T2) | - | - | - | - | - | .789*** | .397*** | .448*** | .417*** | -.043 | -.024 | -.037 | .481*** | .510*** | .498*** |
| PHQ-9  (T3) | - | - | - | - | - | - | .378*** | .443*** | .461*** | -.021 | -.003 | -.039 | .423*** | .413*** | .494*** |
| EDE-  Q-8 (T1) | - | - | - | - | - | - | - | .884*** | .856*** | .011 | .081 | .019 | .292*** | .294*** | .279*** |
| EDE-  Q-8 (T2) | - | - | - | - | - | - | - | - | .910*** | -.046 | .032 | -.036 | .265*** | .310*** | .300*** |
| EDE-  Q-8 (T3) | - | - | - | - | - | - | - | - | - | -.029 | .030 | -0.10 | .225*** | .285*** | .302*** |
| AUDIT  -C (T1) | - | - | - | - | - | - | - | - | - | - | .827*** | .804*** | -.077 | -.059 | -.062 |
| AUDIT  -C (T2) | - | - | - | - | - | - | - | - | - | - | - | .821*** | -.025 | .000 | -.035 |
| AUDIT  -C (T3) | - | - | - | - | - | - | - | - | - | - | - | - | -.055 | -.010 | -.037 |
| Mini-SPIN (T1) | - | - | - | - | - | - | - | - | - | - | - | - | - | .767*** | .713*** |
| Mini-SPIN (T2) | - | - | - | - | - | - | - | - | - | - | - | - | - | - | .757*** |
| Mini-SPIN (T3) | - | - | - | - | - | - | - | - | - | - | - | - | - | - | - |

*Note: N* = 453, T1 = Baseline, T2 = 4 weeks Follow-up, T3 = 8 weeks follow-up, PHQ-9 = 9-item Public Health Questionnaire (module for depression), EDE-Q-8 = 8-item Eating Disorder Examination Questionnaire, Mini-SPIN = Short form of the Social Phobia Inventory, AUDIT-C = Alcohol Use Disorders Identification Test-Consumption, PVSS-21 = 21-item Positive Valence System Scale.

*p < .05. **p < .01. ***p < .001. Two-tailed.

**Table S3** Cross-Lagged Effects of Reward Sensitivity and Depression

|  | *b* | *p* | *SE* | CI |
| --- | --- | --- | --- | --- |
| Model 1: T1, T2, T3 |  |  |  |  |
| RS_T1_ 🡪 D_T2_ | -.054 | .093 | .032 | -0.123, 0.020 |
| RS_T2_ 🡪 D_T3_ | .011 | .732 | .031 | -0.062, 0.074 |
| D_T1_ 🡪 RS_T2_ | -.073 | .039 | .035 | -0.151, 0.005 |
| D_T2_ 🡪 RS_T3_ | -.154 | < .001 | .036 | -0.264, -0.068 |
| Model 1: T1, T3 |  |  |  |  |
| RS_T1_ 🡪 D_T3_ | -.069 | .053 | .036 | -0.140, 0.017 |
| D_T1_ 🡪 RS_T3_ | -.118 | .002 | .038 | -0.210, -0.027 |

*Note*. *N*=453. RS = Reward Sensitivity. D = Depression. Reward Sensitivity was measured with the PVSS-21. Depression was measured with the PHQ-9.

**Table S4** Cross-Lagged Effects of Reward Sensitivity and Social Anxiety

|  | *b* | *p* | *SE* | CI |
| --- | --- | --- | --- | --- |
| Model 1: T1, T2, T3 |  |  |  |  |
| RS_T1_ 🡪 SA_T2_ | -.065 | .034 | .031 | -0.123, -0.002 |
| RS_T2_ 🡪 SA_T3_ | -.055 | .083 | .031 | -0.129, 0.005 |
| SA_T1_ 🡪 RS_T2_ | -.036 | .271 | .033 | -0.101, 0.029 |
| SA_T2_ 🡪 RS_T3_ | -.108 | .001 | .034 | -0.201, -0.030 |
| Model 2: T1, T3 |  |  |  |  |
| RS_T1_ 🡪 SA_T3_ | -.083 | .013 | .034 | -0.144, -0.024 |
| SA_T1_ 🡪 RS_T3_ | -.097 | .007 | .036 | -0.175, -0.025 |

*Note*. *N*=453. RS = Reward Sensitivity. SA = Social Anxiety. Reward Sensitivity was measured with the PVSS-21. Social Anxiety was measured with the Mini-SPIN.

**Figure S1** Cross-Lagged Panel With Reward Sensitivity and Social Anxiety (T1, T2, T3)

**Time 3**

**Time 1**

**Time 2**

**.73*****

**.69*****

Reward Sensitivity

Reward Sensitivity

Reward Sensitivity

**-.06***

1

1

-.05

**-.26*****

**-.06****

-**.05***

1

1

**-.11*****

-.04

Social Anxiety

Social Anxiety

Social Anxiety

**.76*****

**.81*****

*Note.* *N*=453. Standardized path coefficients are reported. Reward Sensitivity was measured with the PVSS-21. Social Anxiety was measured with the Mini-SPIN.

**p* < .05. ***p* < .01 ****p* < .001.

**Figure S2** Cross-Lagged Panel With Reward Sensitivity and Social Anxiety (T1, T3)

**Time 3**

**Time 1**

**.64*****

1

Reward Sensitivity

Reward Sensitivity

**-.08***

Social Anxiety

Social Anxiety

**-.07****

**-.26*****

**-.10****

**.70*****

1

*Note.* *N*=453, Standardized path coefficients are reported. Reward Sensitivity was measured with the PVSS-21. Social Anxiety was measured with the Mini-SPIN.

**p* < .05. ***p* < .01 ****p* < .001.

**Table S5** Cross-Lagged Effects of Reward Sensitivity and Eating Disorder Symptoms

|  | *b* | *p* | *SE* | CI |
| --- | --- | --- | --- | --- |
| Model 1: T1, T2, T3 |  |  |  |  |
| RS_T1_ 🡪 EDS_T2_ | -.035 | .105 | .022 | -0.081, 0.007 |
| RS_T2_ 🡪 EDS_T3_ | -.004 | .844 | .020 | -0.039, 0.047 |
| EDS_T1_ 🡪 RS_T2_ | -.058 | .074 | .033 | -0.111, 0.001 |
| EDS_T2_ 🡪 RS_T3_ | -.101 | .002 | .033 | -0.179, -0.030 |
| Model 2: T1, T3 |  |  |  |  |
| RS_T1_ 🡪 EDS_T3_ | -.026 | .299 | .025 | -0.074, 0.033 |
| EDS_T1_ 🡪 RS_T3_ | -.10 | .005 | .036 | -0.180, -0.021 |

*Note*. *N*=453. RS = Reward Sensitivity. EDS = Eating Disorder Symptoms. Reward Sensitivity was measured with the PVSS-21. Eating Disorder Symptoms were measured with the EDE-Q-8.

**Figure S3** Cross-Lagged Panel With Reward Sensitivity and Eating Disorder Symptoms (T1, T2, T3)

**Time 3**

**Time 1**

**Time 2**

**.72*****

**.70*****

Reward Sensitivity

Reward Sensitivity

Reward Sensitivity

1

1

.00

-.04

-.03

**-.22*****

-.01

1

1

**-.10*****

-.06

**.88*****

Eating Disorder Symptoms

Eating Disorder Symptoms

Eating Disorder Symptoms

**.91*****

*Note.* *N*=453. Standardized path coefficients are reported. Reward Sensitivity was measured with the PVSS-21. Eating Disorder Symptoms were measured with the EDE-Q-8.

**p* < .05. ***p* < .01 ****p* < .001.

**Figure S4** Cross-Lagged Panel With Reward Sensitivity and Eating Disorder Symptoms (T1, T3)

**Time 3**

**Time 1**

**.65*****

1

Reward Sensitivity

Reward Sensitivity

-.03

Eating Disorder Symptoms

Eating Disorder Symptoms

**-.04****

**-.22*****

**-.10****

**.85*****

1

*Note.* *N*=453, Standardized path coefficients are reported. Reward Sensitivity was measured with the PVSS-21. Eating Disorder Symptoms were measured with the EDE-Q-8.

**p* < .05. ***p* < .01 ****p* < .001.

**Table S6** Cross-Lagged Effects of Reward Sensitivity and Alcohol Consumption

|  | *b* | *p* | *SE* | CI |
| --- | --- | --- | --- | --- |
| Model 1: T1, T2, T3 |  |  |  |  |
| RS_T1_ 🡪 AC_T2_ | .031 | .289 | .029 | -0.021, 0.85 |
| RS_T2_ 🡪 AC_T3_ | .009 | .762 | .028 | -0.057, 0.070 |
| AC_T1_ 🡪 RS_T2_ | .06 | .06 | .032 | 0.001, 0.111 |
| AC_T2_ 🡪 RS_T3_ | .038 | .242 | .028 | -0.013, 0.111 |
| Model 2: T1, T3 |  |  |  |  |
| RS_T1_ 🡪 AC_T3_ | .040 | .189 | .030 | -0.029, 0.101 |
| AC_T1_ 🡪 RS_T3_ | .078 | .026 | .035 | 0.012, 0.141 |

*Note*. *N*=453. RS = Reward Sensitivity. AC = Alcohol Consumption. Reward Sensitivity was measured with the PVSS-21. Alcohol Consumption was measured with the AUDIT-C.

**Figure S5** Cross-Lagged Panel With Reward Sensitivity and Alcohol Consumption (T1, T2, T3)

**Time 3**

**Time 1**

**Time 2**

**.73*****

**.72*****

Reward Sensitivity

Reward Sensitivity

Reward Sensitivity

.03

1

1

.01

**.11***

-.03

-.02

1

.06

1

.04

Alcohol Consumption

Alcohol Consumption

Alcohol Consumption

**.79*****

**.80*****

*Note.* *N*=453. Standardized path coefficients are reported. Reward Sensitivity was measured with the PVSS-21. Alcohol Consumption was measured with the AUDIT-C.

**p* < .05. ***p* < .01 ****p* < .001.

**Figure S6** Cross-Lagged Panel With Reward Sensitivity and Alcohol Consumption (T1, T3)

**Time 3**

**Time 1**

**.66*****

1

Reward Sensitivity

Reward Sensitivity

.04

Alcohol Consumption

Alcohol Consumption

-.01

**-.11***

**.08***

**.76*****

1

*Note.* *N*=453, Standardized path coefficients are reported. Reward Sensitivity was measured with the PVSS-21. Alcohol Consumption was measured with the AUDIT-C.

**p* < .05. ***p* < .01 ****p* < .001.

**Figure S7** Mediation Model of Social Anxiety (T1) on Reward Sensitivity (T3)

**Time 3**

**Time 2**

**Time 1**

1

Depression

**b = -.40*****

**a =.47*****

Reward Sensitivity

Social Anxiety

**c = -.27****

1

c’ -.08

*Note.* *N*=453, Standardized path coefficients are reported. Social Anxiety was measured with the Mini-SPIN. Depression was

measured with the PHQ-9. Reward Sensitivity was measured with the PVSS-21.

**p* < .05. ***p* < .01 ****p* < .001.

**Figure S8** Mediation Model of Eating Disorder Symptoms (T1) on Reward Sensitivity (T3)

**Time 3**

**Time 2**

**Time 1**

1

Depression

**b = -.41*****

**a = .44*****

Reward Sensitivity

**c = -.24****

Eating Disorder Symptoms

1

c’ -.06

*Note.* *N*=453, ED = Eating Disorder, Standardized path coefficients are reported. Eating Disorder Symptoms were measured with the EDE-Q-8. Depression was measured with the PHQ-9. Reward Sensitivity was measured with the PVSS-21.

**p* < .05. ***p* < .01 ****p* < .001.

**Figure S9** Mediation Model of Alcohol Consumption (T1) on Reward Sensitivity (T3)

**Time 3**

**Time 2**

**Time 1**

1

Depression

**b = -.43*****

a = -.04

Alcohol Consumption

Reward Sensitivity

**c = .15****

1

c’ = **.13****

*Note.* *N*=453, Standardized path coefficients are reported. Alcohol Consumption was measured with the AUDIT-C. Depression

was measured with the PHQ-9. Reward Sensitivity was measured with the PVSS-21.

**p* < .05. ***p* < .01 ****p* < .001.

**Table S7** Cross-Lagged Effects of Reward Sensitivity and Depression; Subsample of Participants With a History of Depression

|  | *b* | *p* | *SE* | CI |
| --- | --- | --- | --- | --- |
| Model 1: T1, T2, T3 |  |  |  |  |
| RS_T1_ 🡪 D_T2_ | -.031 | .574 | .054 | -0.149, 0.128 |
| RS_T2_ 🡪 D_T3_ | .044 | .473 | .061 | -0.107, 0.156 |
| D_T1_ 🡪 RS_T2_ | -.147 | .019 | .062 | -0.297, -0.020 |
| D_T2_ 🡪 RS_T3_ | -.132 | .087 | .077 | -0.370, 0.079 |
| Model 2: T1, T3 |  |  |  |  |
| RS_T1_ 🡪 D_T3_ | .015 | .816 | .065 | -0.120, 0.164 |
| D_T1_ 🡪 RS_T3_ | -.119 | .113 | .075 | -0.297, 0.027 |

*Note*. *N*=118. RS = Reward Sensitivity. AC = Alcohol Consumption. Reward Sensitivity was measured with the PVSS-21. Alcohol Consumption was measured with the AUDIT-C.

**Figure S10** Cross-Lagged Panel With Reward Sensitivity and Depression (T1, T2, T3); Subsample of Participants With a History of Depression

**.75*****

**.59*****

Reward Sensitivity

Reward Sensitivity

Reward Sensitivity

1

1

.04

-.03

**-.18***

**-.43*****

**-.13***

1

1

-.13

**-.15***

**.80*****

Depression

Depression

Depression

**.80*****

*Note.* *N*=118. Standardized path coefficients are reported. Reward Sensitivity was measured with the PVSS-21. Depression was measured with the PHQ-9.

**p* < .05. ***p* < .01 ****p* < .001.

**Figure S11** Cross-Lagged Panel With Reward Sensitivity and Depression (T1, T3); Subsample of Participants With a History of Depression

**Time 3**

**Time 1**

**.61*****

1

Reward Sensitivity

Reward Sensitivity

.02

Depression

Depression

-.12

**-.28*****

**-.43*****

**.73*****

1

*Note.* *N*=118. Standardized path coefficients are reported. Reward Sensitivity was measured with the PVSS-21. Depression was measured with the PHQ-9.

**p* < .05. ***p* < .01 ****p* < .001.
